# A real-time locating system observes physician time-motion patterns during walk-rounds: a pilot study

**DOI:** 10.1186/1472-6920-14-37

**Published:** 2014-02-25

**Authors:** David R Ward, William A Ghali, Alecia Graham, Jane B Lemaire

**Affiliations:** 1 Department of Medicine, University of Calgary, Health Sciences Center 3330 Hospital Drive NW, Calgary, Alberta T2N4N1, Canada; 2 Institute of Public Health, Community Health Sciences, University of Calgary, Health Sciences Center 3330 Hospital Drive NW, Calgary, Alberta T2N4N1, Canada; 3 W21C Research and Innovation Center, GD01 TRW Building, 3280 Hospital Drive, NW, Calgary, AB T2N4Z6, Canada

**Keywords:** Physicians, Real-time locating system, Teaching rounds, Patients’ rooms, Movement, Workflow

## Abstract

**Background:**

Walk-rounds, a common component of medical education, usually consist of a combination of teaching outside the patient room as well as in the presence of the patient, known as bedside teaching. The proportion of time dedicated to bedside teaching has been declining despite research demonstrating its benefits. Increasing complexities of patient care and perceived impediments to workflow are cited as reasons for this declining use. Research using real-time locating systems (RTLS) has been purported to improve workflow through monitoring of patients and equipment. We used RTLS technology to observe and track patterns of movement of attending physicians during a mandatory once-weekly medical teaching team patient care rounding session endorsed as a walk-rounds format.

**Methods:**

During a project to assess the efficacy of RTLS technology to track equipment and patients in a clinical setting, we conducted a small-scale pilot study to observe attending physician walk-round patterns during a mandatory once-weekly team rounding session. A consecutive sample of attending physicians on the unit was targeted, eight agreed to participate. Data collected using the RTLS were pictorially represented as linked points overlaying a floor plan of the unit to represent each physician’s motion through time. Visual analysis of time-motion was independently performed by two researchers and disagreement resolved through consensus. Rounding events were described as a sequence of approximate proportions of time engaged within or outside patient rooms.

**Results:**

The patient care rounds varied in duration from 60 to 425 minutes. Median duration of rounds within patient rooms was approximately 33% of total time (range approximately 20-50%). Three general time-motion rounding patterns were observed: a first pattern that predominantly involved rounding in ward hallways and little time in patient rooms; a second pattern that predominantly involved time in a ward conference room; and a third balanced pattern characterized by equal proportions of time in patient rooms and in ward hallways.

**Conclusions:**

Observation using RTLS technology identified distinct time-motion rounding patterns that hint at differing rounding styles across physicians. Future studies using this technology could examine how the division of time during walk-rounds impacts outcomes such as patient satisfaction, learner satisfaction, and physician workflow.

## Background

Walk-rounds, a common component of medical education, usually consist of a combination of teaching outside of the patient room as well as in the presence of the patient. The latter is known as bedside teaching and the documented benefits of this method of interacting with patients include improved patient-reported perception of medical teams, increased learner opportunities for development of communication and physical examination skills, and improved attending physician interactions with patients and allied healthcare personnel [1-5]. However, studies have consistently shown decreased utilization rates of these educational techniques in recent decades; from nearly 75% in the 1960’s to 15-30% in more recent literature [6,7]. Potential reasons for this include time constraints due to increased case load and complexity, increased time spent away from the bedside due to reliance on technology such as computerized physician order entry systems to assist with physician workflow, lack of faculty experience and training in how to conduct bedside teaching rounds, and perceived or actual decreased efficiency in daily time-management due to the process of team walk- rounds [8-11].

Much research has focused on ways to improve healthcare workplace efficiency and flow [12-15]. Investigations into physician workflow in particular have previously required direct observation or retrospective surveys, predisposing them to bias [16-19]. Although walk-rounds are inherently a component of physician workflow, there is a paucity of literature documenting the real-time movements of physicians during walk- rounds. Recently, use of novel real-time locating systems (RTLS) technologies to assist in management of patients, supplies, and staff have been advocated to improve healthcare provider workflow, potentially leading to financial and time savings [20]. Our local health authority engaged in related research by installing a RTLS and systematically evaluating its capabilities in locating and tracking items and people as well as the value of this technology to staff [21].

As a pilot sub-study of this work, we sought to use RTLS to observe and track the patterns of movement of staff physicians on an internal medicine Medical Teaching Unit (MTU) during a special Thursday morning rounding period. Thursday afternoons are protected as an academic half-day for all trainees in our residency training program, and as a result, there is a need to complete all or most clinical patient care duties in a compressed half-day that must end at noon. There has been considerable discussion among clinical preceptors and the directorship in our training program regarding the optimal approach to managing patient care in this compressed half day. Because of this, there is special interest in studying rounding patterns on Thursday morning walk-rounds, and more specifically the time-motion activities of various clinical preceptors. The resulting RTLS time-motion profiles give insight into varying staff physician work patterns.

## Methods

### Participants and study design

Our study was an observational study of staff physicians at a single center and represents continuation of previous work [21]. Funding was made possible by “Innovation Toolbox” funding from the Calgary Health Trust. Technology was provided for and calibrated by industry partners IBM and Aero Scout, and data was securely stored on the external Aero Scout mainframe. The funding source and industry partners played no role in the study design, data analysis, results reporting, manuscript preparation, or decision to submit the manuscript for publication.

Eligible participants were staff physicians appointed to a preceptor position on the MTU at the recruitment center and were attending during the study period. Attending physicians may differ widely in the timing, frequency, and style of their patient care rounds based on various factors such as personal preference, day of week, patient load and number of overnight admissions. At our institution, the attending physicians on the MTU are expected to incorporate a mandatory once-weekly medical teaching team rounding session into their schedule, the timing of which is at their discretion. The recommended format for these rounds is that of walk-rounds with bedside teaching. Typically, each attending physician is responsible for the care of 15–20 patients. Physicians perform attending duties for one of two teams in two-week blocks. To enhance continuity, the timing of attending physician-physician team exchange is staggered, so that at any given point in time one physician is performing their first week of duties while the other is performing their second.

Attending physicians on their first week of service were approached during a nine week period from December 2009 to January 2010. As this was a pilot study, statistical analysis to determine study size was not performed. Consecutive on-service preceptors were approached and following an explanation of the RTLS identification system and study objectives, informed consent to participate was obtained. Participants notified the research team in advance of when the rounding session was to occur. Each physician was then provided with an RTLS identification tag to wear during the rounds, and confirmed the start and stop times of each rounding event when they returned the tags to the investigators. The investigators did not follow the attending physicians during the study period and the time-motion data was obtained solely from the RTLS data. A total of nine physicians were approached and eight agreed to participate. We present summary results from the eight participants, with additional detailed pictorial results of four participants in order to portray the identified time-motion profiles. All participants were general internal medicine physicians. Further demographics are withheld in order to preserve confidentiality of the staff physicians. This study was approved by the University of Calgary Conjoint Health Research Ethics Board.

### Setting

**Figure 1:**
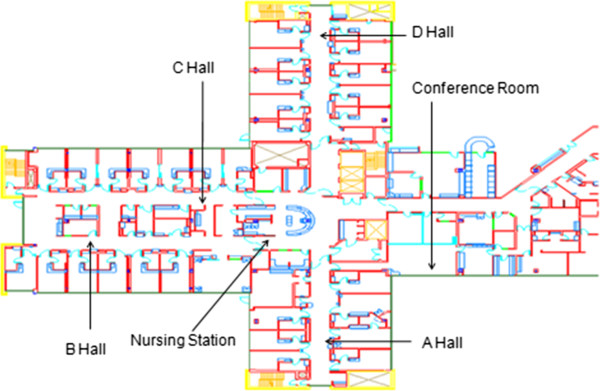
Empty blueprint of MTU with labelled hallways, nursing station, conference room.

The study was conducted on the Ward of the 21^st^ Century (W21C ” see http://www.w21c.org), an acute care medical teaching ward at an urban center in western Canada affiliated with the University of Calgary. The unit also functions as a human laboratory where staff and patients are frequently involved in ongoing research. The physical outline of the unit consists of four hallways that are approximately 2.5 m wide with entrances to patient rooms separated by approximately 3.5 m. Of the 31 patient care rooms on the unit, 28 are single patient rooms, two are double occupancy rooms, and one is a four patient high-observation room. MTU patients may be located in any of these rooms and, if medically necessary or appropriate, or if there are no vacant beds on the MTU, may also be admitted to different wards in the hospital. Additionally, there is a centrally located nursing station and a peripherally located conference room (Figure 1).

### Data collection

We used active Aero Scout mobile view radio-frequency tag models T2 and T3 with EX2000 and EX3200 exciters. These tags emitted a signal at ten second intervals and, via the local wireless fidelity (Wi-Fi) network, data were downloaded to an external mainframe maintained by Aero Scout. Time-location points were superimposed on a blueprint of the unit to form the RTLS. This continually updated system visually represented the location of tags through time as a series of connected dots equally distributed into seven colors in the specific order black, white, grey, light blue, dark blue, teal, and green. For the purposes of visual analysis, segments of time could be selected and represented, to a maximum of 500 time-points, on any single pictograph. In instances where the software separated a single walk-round into multiple pictorials, data sets were combined for the final analysis of each event.

Prior to implementation of the RTLS system for the parent project, Wi-Fi coverage and signal strength were determined to be effective for the space layout. Technologists from Aero Scout and the local health authority verified that the local networks would not interfere with RTLS technology. Tags were approximately the size of a standard identification badge and according to the manufacturer had an ideal accuracy of three meters but in certain cases better results could be achieved. A validation study comparing the RTLS with direct human observation revealed the average error range in location to be approximately two meters [21].

### Data analysis

Two researchers D.W. and A.G. independently reviewed the pictorial representation of each event after data for all events were obtained and the study period ended. Given the margin of error and experience with the RTLS system in the parent study, points located near the edges of hallways or marginally inside rooms were known to represent the tag location in the hallway. Each event was then described as a track through time. Where disagreement between the two researchers with regards to the pattern of movement occurred, consensus was arrived at through discussion. To consolidate visual presentation, we chose to display four individual physician rounding events that exemplified the observed patterns of movement.

## Results

A total of eight physicians were recruited and a total of nine walk-rounding events were recorded. One physician declined participation due to heavy clinical workload. The duration of rounds varied from 60 to 425 minutes with a mean duration of 201 minutes. A median of 33% (range 20-50%) was spent within patient rooms. Increasing round duration did not correlate with increased time within patient rooms.

**Figure 2:**
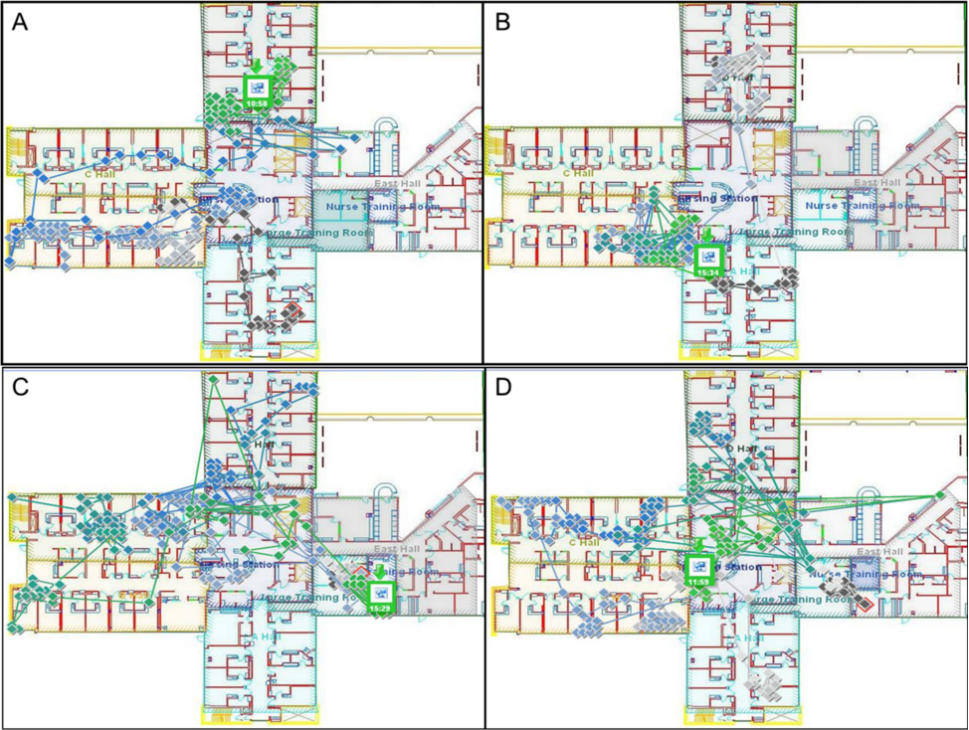
(A-D) Four physician walk-rounding events representative of three time-motion patterns of movement where Panels A and B illustrate hallway rounds predominant pattern, Panel C illustrates conference room rounds predominant pattern, and Panel D illustrates hallway and patient room rounds balanced pattern.

Three distinct physician time-motion activity patterns were observed. These included a pattern that predominantly involved rounding in ward hallways and little time in patient rooms; a second pattern that predominantly involved time in a ward conference room; and a third balanced pattern characterized by equal proportions of time in patient rooms and in ward hallways.We present pictorial data (Figure 2) from four physicians as visual examples of the three patterns of movement as captured by the RTLS. In addition, we supply a description in text as a key supplement to the figures so that the readers can have a better sense of the attending physicians’ movements. Figure 2, Panel A, presents an example of the hallway-predominant pattern of rounding. Dr. 1 first rounded briefly within and outside one room in A hall before s/he moved through B hall, conducted rounds in front of patient rooms, and only briefly entered two patient rooms. Dr.1 then returned to the nursing station before walking B Hall, momentarily entered the previous patient’s room, and continued to D Hall via C Hall. The total duration of this rounding event was 75 minutes and approximately 67% of this time was spent in the hallway. Of the total distance covered during this event, approximately 30% comprised an overlapping path. Dr. 2’s rounding pattern, shown in Figure 2, Panel B, was also considered representative of the hallway-predominant rounding pattern. Dr. 2 began rounding in A Hall and spent half of the total time in this hallway before travelling to D Hall where all rounding occurred in the hall. Subsequently, Dr. 2 travelled to B Hall where the remainder of rounds are comprised of a pattern of three divisions of time between the hallway and the patient care rooms. The total duration of this rounding event was 60 minutes and approximately 67% was spent in the hallway. Of the total distance covered during this event, approximately 40% comprised an overlapping path.Figure 2 Panel C displays Dr. 3’s pattern of movement and is representative of the conference room-predominant rounding pattern that we observed in our data. The initial three hours of these rounds took place within the peripheral conference room. Then, Dr. 3 travelled to a patient room in C Hall. Briefly, Dr. 3 returned to the nursing station prior to returning to the entrance to that previous patient’s room. Dr. 3 then travelled to D Hall and again to C Hall, where rounds were conducted in the hallways before briefly entering one room per hall. A patient in B Hall was seen next with more time spent outside the patient room than inside. The last 90 minutes of rounds concluded in the large conference room. The total duration of this rounding event was 425 minutes and approximately 60% of the time was spent within the conference room. Nearly 50% of the distance travelled comprised a path that overlapped distances previously travelled.Figure 2, Panel D, presents a more balanced rounding pattern seen with only one physician. Dr. 4 began rounds in the large conference room before travelling to A Hall where time was spent exclusively in the hallway. For the remainder of rounds, as patients were seen in B Hall followed by C Hall and finally D Hall, time was spent almost exclusively in patient care rooms. The total duration of this rounding event was 225 minutes, and time was divided equally between the hallway and patient rooms. Of the total distance covered during this event, approximately 30% comprised an overlapping path.

## Discussion

In this pilot-study we used RTLS technology to observe the real-world patterns of movement of attending physicians on an internal medicine service during a mandatory once-weekly medical teaching team patient care rounding session endorsed as a walk-rounds format. The RTLS technology allowed us to map physician movement through time (i.e., a novel use of RTLS technology in the realm of medical education and study of work flow). Analysis was performed on rounding events varying from 60 to 425 minutes in length with a mean duration of 201 minutes. The median proportion of rounds conducted within patient rooms was 33%, and increased total round duration did not correlate with increased time within patient care rooms; findings consistent with previous literature [7,18,19,22]. This pilot study offers a preliminary description of physician time-motion patterns during walk-rounds that may be reflective of distinct rounding styles.

It seems logical that exposure to varied rounding methods throughout a career will lead physicians to adopt those patterns of movement that they feel best allow them to achieve the goals of walk-rounds – namely to enhance clinical education, communication, and physical examination skills through a tradition attributed to Osler [23]. Yet little is known about the optimal combination of patient room/bedside and hallway or classroom teaching during these highly valued rounds. Various authors have suggested methods to improve walk-rounding activities and previous examination of medical learners found that different rounding approaches impact workflow [24-28]. It is therefore possible that varying patterns of movement during walk-rounds also play a role in modulating their efficacy in terms of patient, learner, and attending physician satisfaction.

The RTLS technology offers many opportunities for future research in the realm of medical education and health care delivery. For example, a large number of attending physicians could be observed during informal and scheduled rounding events. Data could be collected varying the day, times of day, patient care loads, and types of learners. There is the additional opportunity to assess efficiency or redundancy of attending physician movement such as how often attending physicians revisit a specific room or retrace their steps. The observed patterns of movement could be linked to outcomes such as patient and learner satisfaction scales in an effort to give insight into the optimal rounding strategy. The RTLS technology could also be used in combination with other research tools such as video ethnography to document what is actually done and discussed in the patient rooms, hallways and conference rooms.

Our study has some limitations. First, it is a small pilot study with limited data collected at a single center and therefore may or may not be generalizable to other centers or non-internal medicine services. Similarly, we identified three time-motion patterns of rounding but, given our small sample size, more could certainly exist. We note that despite a lack of concurrent direct human observation, there was the potential for the Hawthorne effect as physicians may have altered their rounding patterns knowing that they were being tracked via the RTLS system. Additionally, as some MTU patients may be located off of the ward that had the RTLS system, it is possible that segments of events and their corresponding movement patterns could have been missed. We also acknowledge that the inherent variability in the number and acuity of patients, skill of learners, and other factors can alter physician behaviors during walk-rounds. Balancing these points however, is the observed consistency of the proportions of time spent in hallways, the conference room, or patient rooms throughout each event, regardless of the number of patient rooms entered.Additional challenges and limitations of the RTLS technology itself deserve mention. These include an estimated two meter margin of error for system estimates of location and the placement of data points on multiple slides that decreased the clarity and precision of the portrayals of movement (see Figure 2). Fortunately, the study team’s prior experience with the technology from the parent study likely mitigated this challenge. Lastly, although the RTLS technology removed potential observer bias, it did not provide the opportunity to document other factors such as the number of patients visited per event and if bedside teaching actually occurred while attending physicians were at the bedside.

## Conclusions

This small-scale pilot study, undertaken as part of a broader program of work studying the usefulness of RTLS technology within health care systems, allows a preliminary description of attending physician time-motion activity patterns. Our study provides insight into potential physician rounding styles in the special context of a compressed clinical work day in our academic medical center. Our findings derived from RTLS introduce potential relevance of this technology to the field of medical education. In this instance, the technology allowed us to observe and track the patterns of movement of attending physicians during their rounds without observer interference, and three distinct time-motion patterns were identified; more may exist. Future research could explore the reasons for these differences, which rounding approaches are suitable for what types of teaching and learning, which are more acceptable to patients, and other effects of the different approaches on health care delivery and medical education.

## Competing interests

All authors declare they have no competing interests.

## Authors' contributions

DW made substantial contributions to the analysis and interpretation of data, and drafted, critically revised, and provided final approval of the manuscript. WG conceived and designed the study, drafted, critically revised, and provided final approval of the manuscript. AG made substantial contributions to the acquisition, analysis and interpretation of data, and drafted, critically revised, and provided final approval of the manuscript. JL made substantial contributions to the analysis and interpretation of data, and drafted, critically revised, and provided final approval of the manuscript. All authors read and approved the final manuscript.

## Pre-publication history

The pre-publication history for this paper can be accessed here:

http://www.biomedcentral.com/1472-6920/14/37/prepub
